# Assessment of cellular immune phenotype of peripheral blood mononuclear cells in Bangladeshi children with severe acute malnutrition

**DOI:** 10.1016/j.imbio.2025.152887

**Published:** 2025-05

**Authors:** Zannatun Noor, Shaumik Islam, Md. Mehedi Hasan, Ar-Rafi Khan, Md Amran Gazi, Farzana Hossaini, Rashidul Haque, Tahmeed Ahmed, Mustafa Mahfuz

**Affiliations:** a Infectious Diseases Division, International Center for Diarrheal Disease and Research, Bangladesh (icddr,b), Dhaka, Bangladesh; b Nutrition Research Division, International Center for Diarrheal Disease and Research, Bangladesh (icddr,b), Dhaka, Bangladesh

**Keywords:** SAM, PBMC, NK cells, Flowcytometry, Bangladesh

## Abstract

Children suffering from severe acute malnutrition (SAM) have a weakened immune system. The relationship between malnutrition and alterations in the frequency of peripheral blood mononuclear cells (PBMCs) remains unclear. This study investigated the altered immune responses in Bangladeshi children with SAM compared to healthy children. PBMCs were collected from 24 healthy children and 25 children with SAM upon their hospital admission and after 21 days of nutritional therapy at a nutritional rehabilitation unit. Flow cytometry was employed to assess various subsets of T cells, B cells, and natural killer (NK) cells. Children with SAM exhibited significantly lower levels of activated (CD25+) B cells (SAM vs. healthy: 0.18 % vs. 0.30 %, *p* = 0.031) and NK cells (SAM vs. healthy: 4.9 % vs. 9.6 %, *p* = 0.003) compared to healthy controls. Similar immune responses were observed in SAM children during both hospitalization and discharge, with NK cell percentages showing slight increases but remaining significantly lower than in healthy children (SAM endline vs. healthy: 5.9 % vs. 9.6 %, *p* = 0.032). Notable reductions were also observed in CD62+ helper T cells, CD62L+ cytotoxic T cells, and CD62L+ B cells. These results suggest that although SAM children recover clinically, their immune systems remain compromised during discharge.

## 1 Introduction

Severe acute malnutrition (SAM) is one of the foremost global public health problems, with an estimated global caseload of 14 million (Organization, W.H, 2023). Annually, SAM contributes to over a million childhood deaths, presenting a mortality risk approximately 11.6 times higher than that observed among healthy peers (Olofin et al., 2013). The currently accepted definition of SAM by the WHO is very low weight for length/height (below −3z scores of the median WHO growth standards), mid-upper-arm circumference (MUAC) <11.5 cm, or the presence of bilateral pitting edema or both (Organization, W.H, 2012).

SAM manifests through pronounced wasting, nutritional deficiencies, and compromised immune function, often stemming from deficiencies in both micronutrients and energy (Bhutta et al., 2017). Key immune system alterations in severe malnutrition involve T-cell dysfunction and reduced neutrophil microbicidal activity (Bhutta et al., 2017). Various cell signaling cytokines such as tumor necrosis factor-alpha (TNF-alpha), interferon-gamma (IFN-gamma), interleukin-6 (IL-6), and interleukin-10 (IL-10) are activated during infection, facilitating immune cell differentiation (Arango Duque and Descoteaux, 2014). Additionally, in cachexia, the release of interleukin-1 (IL-1), interleukin-6 (IL-6), and tumor necrosis factor (TNF) adversely affects body composition, diminishing appetite, and exerting direct catabolic effects on skeletal muscle and adipose tissue (Rodríguez et al., 2005). This creates a cyclical relationship between acute malnutrition and reduced immune function: Malnutrition heightens infection susceptibility, while infections exacerbate malnutrition by diminishing appetite, promoting catabolism, and intensifying nutrient requirements (Rytter et al., 2014; Tomkins and Watson, 1989). Elevated TNF levels have been linked to increased leptin levels, suppressing hunger and inhibiting overall growth (Dewey and Mayers, 2011; Wong et al., 2010).

Compromised innate immunity results in impaired function of skin and gut epithelial barriers, inhibited granulocyte microbicidal activity, limited circulating dendritic cells, and reduced complement proteins (Bourke et al., 2016). Although malnutrition maintains leukocyte counts and acute phase response, it diminishes delayed-type hypersensitivity responses, decreases soluble IgA in saliva and tears, induces atrophy in lymphoid organs, notably the thymus, and disrupts adaptive immune function (Bourke et al., 2016). Protein-energy malnutrition (PEM) particularly affects the thymus gland, crucial for T-lymphocyte maturation, leading to negative effects on the immature CD4 + CD8+ cell fraction, cell proliferation, and thymic hormone synthesis. Additionally, PEM results in reduced circulating B cells, a shift from Th1 to Th2 cytokines, and lymphocytes with decreased susceptibility to phytohemagglutinin (Prendergast, 2015; Savy et al., 2009).

Severely malnourished children, characterized by impaired immune function and compromised nutritional status, exhibit heightened susceptibility to infections. Even after hospital discharge following conventional in-patient treatment, 25 % of SAM children succumb to mortality in the community over 90 days post-discharge (Kerac et al., 2014). This elevated post-discharge mortality may be attributed to persistent immune compromise in SAM children, unresolved despite malnutrition correction. However, the intricate underlying mechanisms remain poorly elucidated. This study aimed to compare the immune systems of SAM children with healthy counterparts and investigate whether immune disparities persist post-treatment. To gain insights into defense mechanisms, we used peripheral blood mononuclear cells and employed flow cytometry techniques to assess various T cell, B cell, and NK cell subsets in SAM children upon hospital admission and after 21 days of nutritional therapy, as well as in healthy children.

## 2 Methods and materials:

### 2.1 Participants and study design

It was a pilot comparative study where two groups of children were enrolled. One group enrolled 6–24 months children diagnosed with severe acute malnutrition (SAM) just after admission to Dhaka Hospital, icddr,b. Children with SAM met the criteria of having a weight-for-length *Z*-score (WLZ) below -3 and/or mid-upper arm circumference (MUAC) less than 11.5 cm, with or without nutritional edema, and whose parents willingly signed the consent form. SAM children with sepsis or septic shock were excluded. The comparison group includes age-matched healthy children prospectively enrolled from Bauniabadh and adjacent slums of Mirpur, Dhaka. Healthy children were enrolled based on anthropometric scores greater than −2, which encompassed length-for-age z scores (LAZ), weight-for-height z scores (WHZ), and weight-for-age (WAZ) following the WHO growth standards of 2006 (*Organization, W.H. and W.H.O.G.O.f. eHealth, Building foundations for eHealth: progress of Member States: report of the WHO Global Observatory for eHealth*, 2006), and whose parents had signed informed written consent to be included in the study. At the time of recruitment, healthy children were antibiotic and diarrhea-free for the previous 30 days. Additional exclusion criteria for both groups include severe anemia (Hemoglobin <8 g/dl), tuberculosis, other chronic diseases, or any congenital disorder or deformity. This study was approved by the two obligatory components of the institutional review board of the icddr, b: Research Review Committee and the Ethical Review Committee. Written informed consent was obtained from study participant's parents/legal guardians.

### 2.2 Study sites

Children with Severe Acute Malnutrition (SAM) were enrolled from the Dhaka Hospital of the icddr,b, Dhaka, Bangladesh, where the study is being conducted. This hospital provides care and treatment to over 250,000 patients annually, encompassing all ages and both genders. The majority of patients present with diarrheal illnesses and/or other associated conditions such as pneumonia, malnutrition, sepsis, and electrolyte imbalances. Most of the patients come from low socioeconomic backgrounds and reside in urban and peri-urban areas of Dhaka. A multidisciplinary team of junior and consultant physicians, nurses, counselors, and dietary workers provides care. The hospital is equipped with specialized wards for treating respiratory issues, diarrheal diseases, and malnutrition. Healthy children were enrolled from the Bauniabadh slum area of Mirpur, Dhaka, a densely populated settlement characterized by low socioeconomic status and inadequate sanitary conditions. Detailed information regarding the study site, geography, and sociodemography has been published elsewhere (Barratt and Gordon, 2017).

### 2.3 Management protocol of SAM in Dhaka Hospital

All children admitted with SAM are initially treated for the acute phase in the Longer Stay Unit according to the standardized treatment protocol for SAM children at icddr,b (Ahmed et al., 1999). After recovering from acute illness and stabilization, the infants were transferred to the Nutritional Rehabilitation Unit (NRU) at icddr,b. During the acute phase, therapeutic milk (F-75; WHO protocol) was administered. In the NRU, F-75 was replaced by F-100 therapeutic milk (WHO protocol). The initial ‘acute phase’ treatment aimed to manage complications arising from the acute illnesses, followed by the ‘rehabilitation phase’ to achieve catch-up growth with a focus on diet. Both feeds were prepared by the hospital dietician, with F-100 administered every 2–3 h at a daily volume of 150 ml/kg for non-edematous infants and 130 ml/kg for edematous infants. Additionally, caregivers at the NRU received routine sessions on psychosocial stimulation, follow-up care, and other related topics. At the NRU, vaccination is provided to children who have missed any doses according to the age-specific Expanded Program on Immunization (EPI) schedule in Bangladesh. Breastfeeding was encouraged between feeds.

**Fig. 1:**
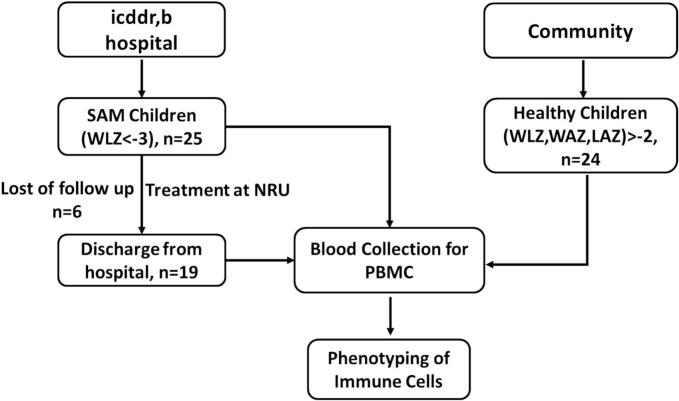
Flow diagram of research methodology.

Written informed consent, obtained in compliance with the icddr,b Institutional Review Board approval, was provided by all study participants. All methods were performed in accordance with the relevant guidelines and regulations. Children with SAM received 21 days of nutritional intervention at the Nutrition Rehabilitation Unit (NRU) of icddr,b hospital. Blood samples were collected from SAM children at two different time points: a) at the time of admission (baseline), and b) at the time of hospital discharge (endline). The blood samples from healthy children were drawn at the moment of enrollment (Fig. 1).

### 2.4 Data and sample collection

During the enrollment process, socio-economic, anthropometric and demographic information was gathered from all study participants by a proficient nurse and field staff 5 ml of venous blood in a sodium heparin tube was collected ensuring proper handling and transportation to the Parasitology laboratory at icddr,b while maintaining the necessary cold chain.

### 2.5 Laboratory analysis

#### 2.5.1 Isolation of Peripheral Blood Mononuclear cells and storage

The whole blood was centrifuged at 4000 rpm for 10 min to separate plasma. After separating the plasma, the remaining blood cells were diluted 1:1 using R-10 media (89 % Roswell Park Memorial Institute (RPMI) 1640 (Gibco, Grand Island, NY, USA), 10 % fetal bovine serum (FBS; Gibco), and 1 % penicillin–streptomycin (Gibco) which was finally used for peripheral blood mononuclear cell (PBMC) separation. PBMC was isolated by density gradient centrifugation method using Ficoll-Paque plus (Cytiva, Marlborough, MA, USA). Isolated PBMC were cryopreserved in liquid nitrogen using the cryoprotectant (90 % FBS and 10 % dimethyl sulfoxide (DMSO) in cryovials prior to further analysis.

#### 2.5.2 PBMCs staining and flowcytometry

Cryopreserved PBMCs were thawed and the immune cells were phenotyped using a flow cytometer. To minimize batch effects, both the SAM baseline and endline samples, as well as the healthy control PBMCs, were processed together in the same run. Thawed PBMCs were handled with great care, suspended in 1 ml of R10 media (containing 10 % FBS in RPMI, 1 % penicillin-streptomycin, 1 % HEPES, 1 % non-essential amino acids, and 1 % sodium pyruvate), and thoroughly mixed. The thawed PBMCs were then allowed to rest at room temperature for 3 h to enable the desired cluster of differentiation (CD) marker expression on the cells.

After the cell resting period, 2 μl of Fc-block solution was added, followed by a 10-min incubation on ice. Subsequently, live/dead staining was carried out, and then 1 ml of staining buffer solution (containing 5 % FBS in PBS) was added to wash away the unbound Fc-block and live/dead staining dye. Fluorochrome-tagged antibodies CD3-PereCP, CD19-PE, CD4-APC-Fire 750, CD8-FITC, CD45-BV 510, CD25-PE Dazzle, CD16-PE-Cy7, CD 62 L-APC, CD56-PE-Cy7 were used to stain PBMCs, antibodies details listed in supplementary table 1. The antibody panel's master mix was added to the cells and incubated for 30 min on ice. Afterward, staining buffer solution was added to remove any unbound antibodies, followed by the addition of another 300 μl of stain buffer solution to resuspend the stained cells before transferring them to the FACS tube for analysis in the flow cytometer (FACS Aria III). The flow cytometer was calibrated before each run. Compensation controls and fluorescence minus one (FMO) sample (Supplementary fig. 1) were used to ensure accurate data and create appropriate gating strategies (Supplementary fig. 2). Data were acquired using FACSdiva software, and data analysis was performed using OMIQ software. All calculations were based on cell proportions relative to the parent gate. List of all cells population that were quantified as a percentage of the indicated parent population in the panel are given in supplementary table 2.

##### 2.5.2.1 **Sample size calculation**

The sample size was calculated based on Najera et al. 2004 (Nájera et al., 2004). Using flow cytometry, they examined the peripheral-lymphocyte subsets between well-nourished and malnourished children aged 6–29 months. They observed that the proportion of B lymphocytes (CD20^+^) in malnourished was lower than that seen in well-nourished children (22.8 % versus 31.9 %, *P* < 0.05). The estimated sample size was 23 in each group considering an attrition rate of 20 %. It was calculated based on the results of this published study for the mean percentage values of B lymphocytes present in children with SAM and healthy children with a 5 % level of significance and 80 % power for comparing two means. However, without attrition, each group's calculated sample size was 19 (Table 1).

**Table 1:** Sample size calculation.

Indicators	Information
Group ‘A' mean, m1	31.90
Group ‘B' mean, m2	22.80
Standard Deviation, σ	10.00
Level of significance (α)	0.05
Power (1-β)	0.80
Z alpha value	1.96
Z beta value	0.84
Sample size for group-1 (n1)	19.0
Sample size for group-2 (n2)	19.0
Sample size for both group (n1 + n2)	38
Attrition (%)	20 %
Total Sample Size (with attrition)	48

### 2.6 Statistical analysis

In the data summary, mean with standard deviations were used for quantitative symmetric variables, while quantitative asymmetric variables were summarized using median values with the first quartile and third quartile. Fisher exact test or chi-square test were used to evaluate categorical variables. The Wilcoxon matched-pairs signed rank test was used to compare data between the children with SAM at baseline) and endline. The Mann–Whitney *U* test was utilized to compare the data of healthy children with that of children with SAM (baseline) at hospital admission or children with SAM (endline) at hospital discharge. A *p*-value less than 0.05 was considered the threshold for statistical significance in all analyses, and all statistical procedures were carried out using STATA version 15.

## 3 Results

### 3.1 Socio-demographic and clinical characteristics

The study enrolled 49 participants, comprising 25 SAM children 24 healthy children. First recruitment date of this study was on 4th March 2020 and the end of the recruitment was on 10th March 2021. Children with SAM had a mean age of 13.2 ± 4.6 months, while control children were 14.4 ± 5.6 months. Of the participants, 36 % were female among SAM children, and 45.8 % were female in the control group. Age and sex did not significantly differ among the participants (Table 2).

**Table 2:** Comparison of demographic and anthropometric status between SAM and healthy children at enrollment.

Indicators	SAM (*n* = 25)	Healthy (*n* = 24)	p-value
Child age in, Mean ± SD	13.2 ± 4.6	14.4 ± 5.6	0.416
Female children, n (%)	9 (36)	11 (45.8)	0.484
Weight in kg, Mean ± SD	6.2 ± 1	9.8 ± 1.9	<0.001
Length in cm, Mean ± SD	69.8 ± 5.8	76.4 ± 7	<0.001
WLZ, Mean ± SD	−3.4 ± 0.3	0.1 ± 0.7	<0.001
LAZ, Mean ± SD	−2.5 ± 1.3	−0.1 ± 0.7	<0.001
WAZ, Mean ± SD	−3.8 ± 0.7	0.04 ± 0.7	<0.001
Family income in USD, Median (IQR)	212.3 (141.5, 294.8)	153.3 (117.9, 235.8)	0.118
H/O exclusive breast feeding up to 6 Months, n (%)	13 (52)	6 (25)	0.072
Treatment of drinking water, n (%)	8 (32)	19 (79.2)	0.001
Always hand wash before preparing food, n (%)	4 (16)	8 (33.3)	0.470
Always hand wash after using toilet, n (%)	21 (84)	20 (83.3)	1.00
Maternal antibiotic usage, n (%)	14 (56)	5 (21)	0.012^1^
Child antibiotic usage, n (%)	21 (84)	0 (0)	
Commonly used antibiotic (Azithromycin), n (%)	15 (71)	–	
Duration of antibiotic in days, Median (IQR)	2 (1, 5)	–	

**Footnote:** The P-value for the difference was determined by *t*-test for continuous data and the chi-square or Fisher's exact test for the categorical data. Values are expressed as mean ± SD. HAZ = Height-for-age z score; WAZ = Weight-for-age z score; WHZ = weight-for-height z score.

However, there were significant differences between SAM children and healthy participants in terms of WLZ, LAZ, and WAZ scores. The mean (±SD) WLZ was −3.4 ± 0.3 for SAM children and 0.1 ± 0.7 for healthy children (*p* value <0.001). Furthermore, the mean (±SD) LAZ was −2.5 ± 1.3 for SAM children and − 0.1 ± 0.7 for healthy children (p value <0.001). WAZ was −3.8 ± 3.8 for SAM children and − 2.5 ± 1.0 for healthy children (p value <0.001) (Table 2).

Family income and exclusive breastfeeding rates were higher in the SAM group compared to the healthy control group, which was an unexpected finding. Recent data from the Bangladesh Demographic and Health Survey (Hossain et al., 2022) indicate that breastfeeding rates and family income are typically lower among severely wasted children. However, the sample size in this study was not sufficiently powered to detect statistically significant differences in these variables. It is also important to note that the control group comprised healthy children from a slum in Dhaka, who, despite their relatively better nutritional status, had lower rates of exclusive breastfeeding and family income. In contrast, many of the SAM children admitted to the hospital were not residents of slum areas and exhibited higher exclusive breastfeeding rates and family income levels.

Out of the 25 SAM children, 19 successfully completed a 21-day nutritional treatment at the Nutrition Rehabilitation Unit (NRU) department of icddr,b hospital. The mean WLZ score for SAM children (*n* = 25) increased at the time of discharge from the hospital (*n* = 19) after 21 days of nutritional treatment compared to their admission (*P* value = 0.033) (see Table 3). However, there were no significant differences in LAZ and WAZ between hospital admission (baseline) and after 21 days during hospital discharge (endline) for children with SAM (Table 3).

**Table 3:** Comparison of baseline and endline anthropometric condition of SAM children.

**Indicators**	**Baseline (n** **=** **25)**	**Endline (n** **=** **19)**	**p-value**
WLZ at enroll, Mean ± SD	−3.4 ± 0.3	−3.1 ± 0.7	0.033
LAZ at enroll, Mean ± SD	−2.5 ± 1.3	−2.8 ± 1.4	0.459
WAZ at enroll, Mean ± SD	−3.8 ± 0.7	−3.6 ± 1.0	0.638

### 3.2 Phenotypic distribution of peripheral mononuclear cells in children with SAM during hospital admission compared to healthy children

**Fig. 2:**
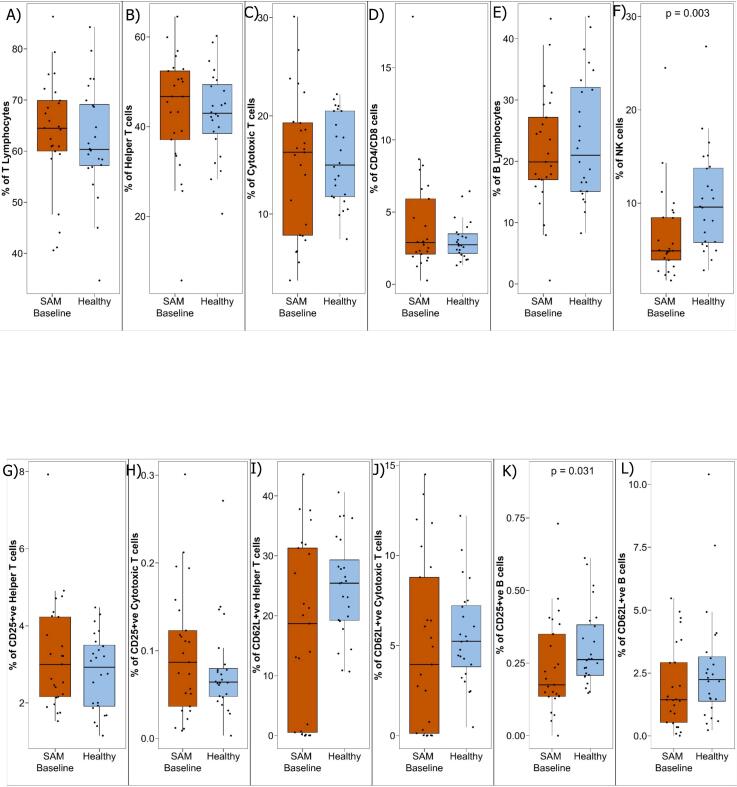
Phenotyping of PBMCs in children with SAM (baseline) and healthy children. Blood samples were collected from children and PBMCs were isolated to identify different T lymphocytes, B lymphocytes and NK cells subsets. PBMCs run on flowcytometry to phenotyping cellular distribution in children with SAM and healthy children.

We conducted a phenotypic analysis of stored PBMC to observe different subsets of T cells, B cells, and NK cells among children with SAM and healthy children. Our analysis revealed no statistically significant differences in the frequencies of T cells, helper T cells, and cytotoxic T cells, B cells between SAM and healthy children (Fig. 2A-2E). In addition, CD25 positive Th Cells, CD25 positive Tc Cells, CD62L positive Th Cells, and CD62L positive Tc Cells were not significantly different (Fig. 2G-2J). However, CD25 positive B cells were decreased in children with SAM at the time of enrollment (baseline) compared to healthy children (*p* value = 0.031) (Fig. 2K). Significant differences were not found between the children with SAM and healthy children for CD62L positive B cells (Fig. 2L). However, there is significant reduction of NK cells in children with SAM at enrollment (baseline) compared to healthy children (*p*-value = 0.0031) (Fig. 2F). The percentage of NK cells and CD25 positive B cells are positively correlated with the nutritional status LAZ, WAZ, WLZ, however CD25 negative B cells are negatively correlated (Table 4).

**Table 4:** Correlation of lymphocyte subsets with nutritional LAZ, WAZ and WLZ status.

Cells name	LAZ	WAZ	WLZ
CD3+ cells	−0.08	−0.17	−0.14
CD25 + CD3 + cells	0.09	0.09	0.03
NKT cells	0.11	0.12	0.08
CD62L+ T cells	0.21	0.17	0.03
Tc cells	0.01	0.04	−0.04
CD62L+ Tc cells	0.19	0.14	0.00
CD4 + CD8+ cells	0.02	−0.08	−0.07
Th cells	−0.04	−0.07	0.02
CD62L+ Th cells	0.21	0.18	0.04
B cells	−0.10	−0.09	−0.07
CD25+ B cells	0.37*	0.40*	0.32*
CD25- B cells	−0.35*	−0.39*	−0.33*
NK cells	0.46*	0.44*	0.34*

Spearman correlation with a 0.05 level of significance. * indicating <0.05.

### 3.3 Nutritional therapy did not make changes in cellular distribution in children with SAM

**Fig. 3:**
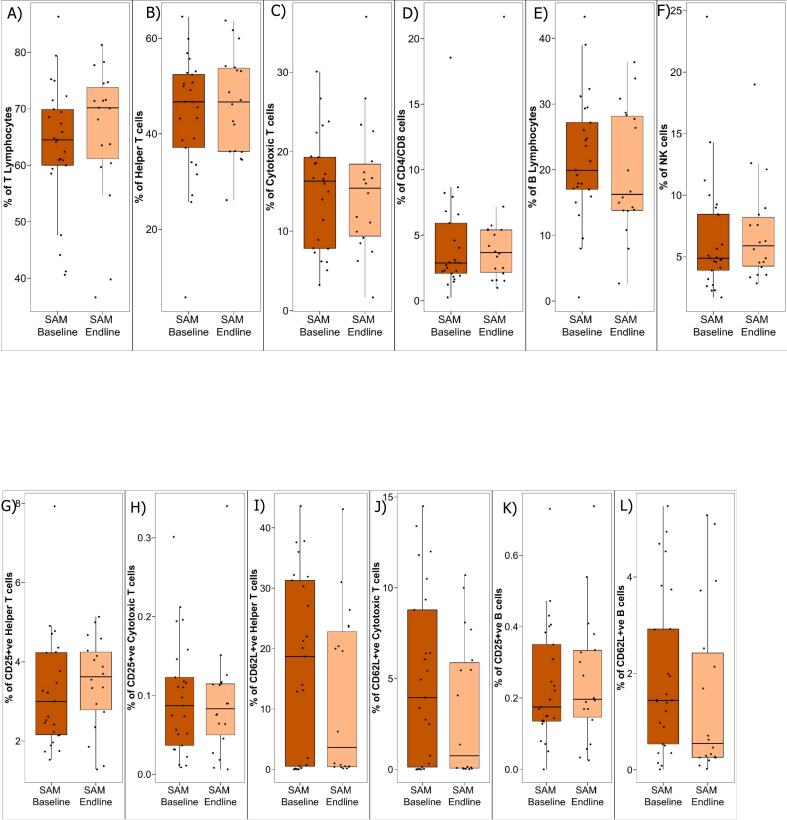
Phenotyping of PBMCs in children with SAM at hospital admission (baseline) and at hospital discharge (endline). Blood samples were collected from children and PBMCs were isolated to identify different T lymphocytes, B lymphocytes, and NK cell subsets. PBMCs run on flow cytometry to phenotyping cellular distribution in children with SAM at their hospital admission (baseline) and at their hospital discharge (endline).

There was no significant phenotypic cellular distribution of helper T cells, cytotoxic T cells, ratio of helper and cytotoxic T cells, B cells, NK cells, CD25 positive Th Cells, CD25 positive Tc Cells, CD62L positive Th cells, CD62L positive Tc cells, CD25 positive B cells and CD62L positive B cells between children with SAM at their hospital admission (baseline) and at their hospital discharge (endline) (Fig. 3A-3L).

### 3.4 Phenotypic distribution of peripheral mononuclear cells in children with SAM during hospital discharge compared with healthy children

**Fig. 4:**
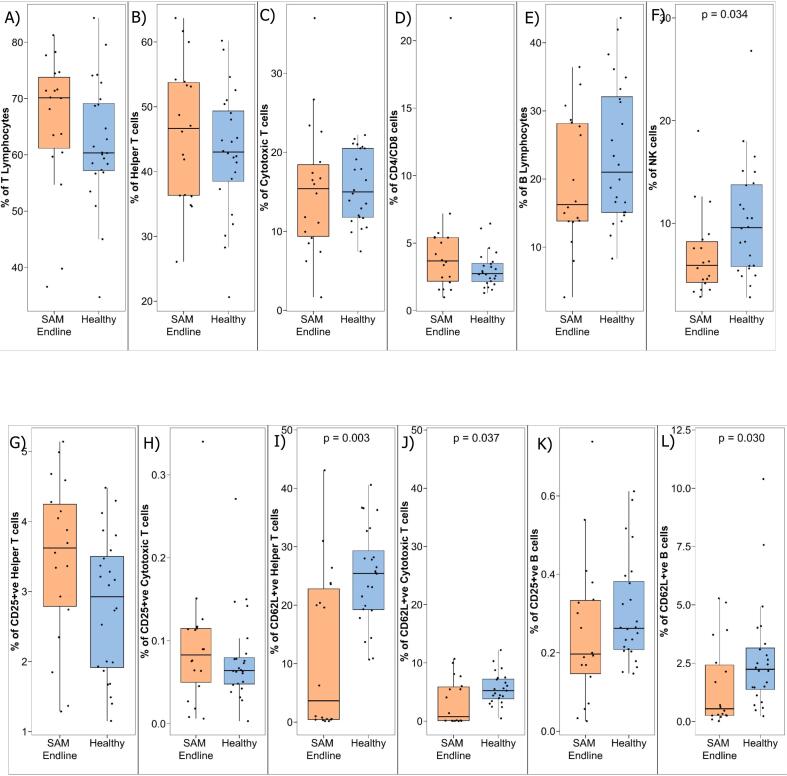
Phenotyping of PBMCs in children with SAM at their hospital discharge (endline) and healthy children. Blood samples were collected from children and PBMCs were isolated to identify different T lymphocytes, B lymphocytes and NK cells subsets. PBMCs run on flowcytometry to phenotyping cellular distribution in children with SAM at their hospital discharge (endline) and healthy children.

There were no statistically significant differences in helper T cells, cytotoxic T cells, ratio of helper and cytotoxic T cells and B cells (Fig. 4A-4E). Significant reduction was observed in NK cells of children with SAM at their hospital discharge (endline) compared to healthy children (*p*-value = 0.0034) (Fig. 4F). CD25 positive Th Cells, and CD25 positive Tc Cells, CD25 positive B cells did not show differences between children with SAM at their hospital discharge (endline) and healthy children (Fig. 4G-4H, 4K).

However, CD62L expressing helper T cells (*p* value = 0.003) (Fig. 4I), CD62L expressing cytotoxic T cells (p value = 0.037) (Fig. 4J) and CD62L expressing B cells (p value = 0.03) (Figure: 4 L) are decreased in children with SAM at the time of discharge from the hospital (endline) compared to healthy children.

## 4 Discussion

Underweight, wasting, and stunting resulting from inadequate food and micronutrient intake can, at times, coincide with infections causing weight loss, ultimately resulting in severe acute malnutrition (SAM). Infants are particularly susceptible to this condition during their growth and development (Kebede et al., 2022). There is a dearth of information regarding the immunological changes linked to SAM, especially among Bangladeshi children (Noor et al., 2023). SAM and co-infections can impact both adaptive and innate immunity, though the exact mechanisms remain unclear due to scarce of data. The findings from our current study indicate that T lymphocyte cells and T cell subsets do not exhibit significant differences in children with SAM when compared to a healthy group of children. Previous research documented that malnourished population have alterations in the frequency of CD4+ and CD8+ T lymphocytes (Marcos et al., 1993). A separate study on protein-energy malnutrition (PEM) found decreased level of thymic hormone and a reduction in CD4+ and CD8+ T cell proliferation due to an abnormality in the maturation of these cell subsets in the thymus (Savy et al., 2009). However, no differences were observed in CD4+ T cells, CD8+ T cells, or the CD4+ T cells to CD8+ T cells ratio between children with SAM and the healthy cohort in our study.

In the current study, B cell percentages were alike in both children with SAM and their healthy counterparts. However, SAM children displayed a notable reduction in CD25+ B cells during their enrollment and in comparison, to the healthy control group. CD25 positive B cells are considered as activated B cells (Brisslert et al., 2006). Noor et al. demonstrated an increase in CD25-positive B cells following supplementation in the cohort at risk of stunting. This finding implies that nutritional intervention may influence CD25+ B cell levels among children who are at risk of stunting (Noor et al., 2023). Our research also unveiled lower levels of NK cells in children with SAM compared to their healthy counterparts. The innate immune system relies significantly on NK cells for exerting its function (Mandleboim, 2000). The diminished NK cell count in children with SAM could lead to functional impairments and potentially result in increased anti-inflammatory responses or reduced pro-inflammatory cytokine production.

In a recent study, a decrease in CD3+, CD4+, and CD8+ cell counts were observed in well-nourished children infected with bacteria when compared to well-nourished non-infected children (Nájera et al., 2004). The host's nutritional state can affect the immune system's ability to function properly, which in turn influences how the host responses to infections (Beisel, 1979). Children with SAM experience severe deficiencies in energy, protein, and vital nutrients (including vitamins A, C, E, D, and zinc), which weakens their immune systems and heightens their vulnerability to infections (Dipasquale et al., 2020). Malnutrition affects both the innate and adaptive immune systems, diminishing an individual's ability to effectively combat diseases and respond to vaccinations (Chandrasekaran et al., 2017).

We examined the immunological characteristics of children with SAM upon hospital discharge and discovered that the levels of CD62L positive T cells and CD62L positive B cells are significantly reduced in comparison to healthy children. This difference persists even after the SAM children have received 21 days of nutritional treatment at the hospital. It may because of the disturbance in the expression of adhesion molecules, including CD62L, in the children with SAM which can affect the homing and localization of immune cells (Wirth et al., 2009). In children with SAM at their hospital discharge, we observed lower levels of T cell subsets, including CD62L positive helper T cells and CD62L positive cytotoxic T cells, in comparison to the control group. This suggests a compromised immune system in children with SAM upon their discharge from the hospital. In the case of SAM, T cell subsets like helper T cells and cytotoxic T cells could exhibit similar percentages compared to the healthy cohort. However, what sets them apart is possibly because of the absence of activated T cells required to establish an environment for combating adverse conditions. Our study revealed reduced CD62L expression in children with severe acute malnutrition (SAM), likely due to chronic immune activation driven by malnutrition, which promotes the differentiation of naïve T cells into effector and memory T cells. This process involves the shedding of CD62L to enable T cell migration to peripheral tissues. A similar mechanism is observed in cytomegalovirus (CMV) infection, where immune activation leads to CD62L modulation and shedding, highlighting how persistent inflammation and stress in SAM may parallel immune responses seen in CMV infection, contributing to diminished CD62L levels (Hertoghs et al., 2010; Zieliński et al., 2017). Studies have indicated that the expression of CD62L on CD8 T cells is linked to an increased quantity of effector and memory T cells in lymph nodes, whereas the absence of CD62L causes these cells to be excluded from lymph nodes (Yang et al., 2011). The regulation of CD62L is crucial for regulating T cell flow to and from peripheral lymph nodes (Yang et al., 2011). Furthermore, the decreased levels of NK cells persist even after a 21-day nutritional intervention in children with SAM when compared to healthy children. From our study, we were unable to determine why children with SAM continue to exhibit dysregulated immune function at the time of hospital discharge (endline) despite receiving nutritional therapy. It's possible that different nutritional therapies and an extended duration or different dosing may be required to witness an improvement in the immune status of these children with SAM. Additionally, there is limited data available regarding long-term outcomes following discharge from SAM treatment, particularly with respect to the immune cellular status. After being discharged from the hospital, children recovering from SAM continue to experience infectious morbidity and mortality (Chisti et al., 2014). Post-discharge mortality was observed at a rate of 8.7 % among severely malnourished children with pneumonia infection, within a group of 369 Bangladeshi children within 3 months of their hospital discharge (Chisti et al., 2014). A study on children with SAM in Malawi revealed a high post-discharge mortality rate after treatment from the hospital. The study indicated that 42 % of children with SAM died during or after treatment, with 25 % of these deaths occurring after being discharged from the regular program (Kerac et al., 2014). Hence, there is a pressing need for enhanced treatment guidelines for SAM. Immune cell modulation-directed nutritional therapy could prove more effective in enhancing the nutritional and immunological status of children with SAM. For example, zinc might play a role in increasing the number and function of NK cells. Research has demonstrated that zinc supplementation significantly increases the number of cells and facilitates the differentiation of CD34+ progenitor cells into NK cells (Muzzioli et al., 2009). In vivo studies have shown that zinc supplementation leads to a greater quantity of IFN-g-producing NK cells (Muzzioli et al., 2009). Moreover, iron has been found to impact the activation and differentiation of various subtypes of T cells and NK cells, as well as antibody responses in B cells (Ni et al., 2022). A study conducted in Bangladesh reported an increase in NK cell numbers in healthy men after receiving vitamin A supplementation (Ahmad et al., 2009). In our study, after 21 days nutritional therapy the WLZ score increased among children with SAM by discharge, however, they were still significantly lower than healthy controls. This result suggesting the need of continued nutritional support to children with SAM to achieve a healthy status. Study conducted in Bangladesh also shown that supplementation with microbiota-directed complementary food prototype (MDCF-2), provided to children with moderate acute malnutrition revealed an increase levels of plasma proteins, including those involved in bone growth and neurodevelopment (Chen et al., 2021). Consequently, there is a growing need to place greater emphasis on nutritional therapy that can contribute to the enhancement of immune cellular status.

In our study, we did not have the opportunity to monitor the immune profile of SAM children after their discharge from the hospital, particularly when they had fully recovered from the infection. We also missed the chance to identify any patterns in immune cell response during their recovery. The study has several shortcomings for instance, children with SAM were recruited from hospital settings, whereas the healthy control group was enrolled from the community. While we observed a similar type of T cell response in both cohorts, we did not differentiate between active and naive T cells. Furthermore, we did not compare the T cell and B cell subset response with their functional response, such as cytokine production capacity with different stimuli or cytokines level in serum between children with SAM and healthy children. This study did not investigate the mucosal response, impact of antibiotics and the role of microbiome in SAM children. The pathogen status of the children was not identified is also another limitation of the study.

In summary, this was the initial investigation into comparing the immunological profiles of SAM children and compared it with healthy children. The study yielded important findings, indicating that crucial immune cells, including NK cells and CD25-positive B cells, are diminished in children with SAM. Additionally, during the hospital discharge of children with SAM, there is also a decrease in the levels of CD62L-positive B cells, cytotoxic T cells, and helper T cells. The guidelines for follow-up care for SAM children should be reevaluated to enhance their health outcomes and reduce post-discharge mortality rates.

A longitudinal study, with data collected at multiple time points, could help unveil patterns in the immune profiles of SAM children. Future avenues may include single-cell analysis and translating findings into clinical practice. Additionally, by aggregating data related to the SAM algorithm, it may be possible to establish patterns in immune cell behavior using artificial intelligence, leading to the identification of novel biomarkers for SAM in children.

## Authors Contribution

MM and TA originated the idea for the study and led the protocol. ZN and MH developed the methodology. MH and FH took part in laboratory analysis. MM, ZN, SI conceptualized the manuscript. ZN, SI, AK, MH planned the data analysis and interpretation. SI, MH did the literature review. MM and AG supervised the work. MM, TA and AG critically reviewed the manuscript. All authors have read and approved the final draft of the manuscript.

## Funding

This work was supported and funded by the Bill & Melinda Gates Foundation (BMGF) under its Global Health Program. The project investment ID is OPP1136751.

## Ethics Statement

The protocol was approved by the Research Review Committee and Ethical Review Committee of icddr,b (approved protocol number 19092, date: 25 February 2020). All procedures were performed in compliance with relevant laws and institutional guidelines and have been approved by the IRB. Consent obtained from parent(s)/guardian(s) and the privacy rights of human subjects have been observed.

## CRediT authorship contribution statement

**Zannatun Noor:** Writing – review & editing, Writing – original draft, Supervision, Project administration, Methodology, Investigation, Formal analysis, Data curation, Conceptualization. **Shaumik Islam:** Writing – review & editing, Methodology. **Md. Mehedi Hasan:** Writing – review & editing, Methodology. **Ar-Rafi Khan:** Writing – review & editing, Formal analysis, Data curation. **Md Amran Gazi:** Writing – review & editing, Methodology, Investigation. **Farzana Hossaini:** Writing – review & editing, Methodology. **Rashidul Haque:** Writing – review & editing, Supervision, Investigation. **Tahmeed Ahmed:** Writing – review & editing, Visualization, Supervision, Resources, Investigation, Funding acquisition, Conceptualization. **Mustafa Mahfuz:** Writing – review & editing, Validation, Resources, Project administration, Investigation, Funding acquisition, Formal analysis, Conceptualization.

## Declaration of competing interest

The authors declare that they have no known competing financial interests or personal relationships that could have appeared to influence the work reported in this paper.

## Data Availability

The data in this study are available from the corresponding author on reasonable request.

## Appendix A Supplementary data

Supplementary material **Supplementary fig. 1:** Florescence minus one (FMO) gating of peripheral blood mononuclear cells (PBMCs). FMO gating strategy used to identify cells population, like, CD45 positive cells, T lymphocytes (CD3 positive), helper T cells (CD)4 positive, cytotoxic T cells (CD8 positive), B cells (CD19 positive), CD56 and CD16 positive NK cells, CD25 positive helper T cells, CD25 positive cytotoxic T cells, CD62L positive helper T cells, CD62L positive cytotoxic T cells, CD25 positive B cells and CD62L positive B cells. **Supplementary fig. 2:** Gating strategy of peripheral blood mononuclear cells (PBMCs) on flowcytometry analysis. to phenotyping different cells. Gating strategy used to identify cells population, like, live and dead cells, CD45 positive cells, T lymphocytes (CD3 positive), helper T cells (CD)4 positive, cytotoxic T cells (CD8 positive), B cells (CD19 positive), CD56 and CD16 positive NK cells, CD25 positive helper T cells, CD25 positive cytotoxic T cells, CD62L positive helper T cells, CD62L positive cytotoxic T cells, CD25 positive B cells and CD62L positive B cells. **Supplementary table 1:** Antibody panel for phenotyping **Supplementary table 2:** List of all cells population that were quantified as a percentage of the indicated parent population in the panel. Supplementary material
