# Supplementary material for: Assessment of cellular immune phenotype of peripheral blood mononuclear cells in Bangladeshi children with severe acute malnutrition
Source: Immunobiology. 2025 May;230(3):152887. doi: 10.1016/j.imbio.2025.152887 (PMC12137146; doi:10.1016/j.imbio.2025.152887)

**Supplementary table 1:** Antibody panel for phenotyping

| SN | Marker | Fluorochrome | Clone | Company |
| --- | --- | --- | --- | --- |
| 1 | Live/Dead | (Fixable Near-IR) | - | Thermo Fisher |
| 2 | CD3 | PerCP | UCHT1 | Biolegend USA |
| 3 | CD19 | PE | SJ25C1 | Biolegend USA |
| 4 | CD4 | APC-Fire 750 | SK3 | Biolegend USA |
| 5 | CD8 | FITC | RPA-T8 | Biolegend USA |
| 6 | CD45 | BV 510 | HI30 | Biolegend USA |
| 7 | CD25 | PE Dazzle | M-A251 | Biolegend USA |
| 8 | CD16 | PE-Cy7 | 3G8 | Biolegend USA |
| 9 | CD62L | APC | DREG-56 | Biolegend USA |
| 10 | CD56 | PE-Cy7 | HCD56 | Biolegend USA |

**Supplementary table 2:** List of all cells population that were quantified as a percentage of the indicated parent population in the panel

| Parent population | % gated |
| --- | --- |
| CD45 +ve cells | CD3+ ve lymphocytes (T cells) |
| CD3+ ve lymphocytes | CD4+ ve (Helper T cells) |
| CD3+ ve lymphocytes | CD8+ ve (Cytotoxic T cells) |
| CD3+ ve lymphocytes | CD4/CD8 T cells |
| CD3-ve cells | CD19+ve (B lymphocytes) |
| CD3-ve cells | CD16 CD56 (NK cells) |
| CD3+ve CD4+ve | CD25+ ve cells (CD25+ve Helper T cells) |
| CD3+ve CD8+ve | CD25+ ve cells (CD25+ve Cytotoxic T cells) |
| CD3+ve CD4+ve | CD62L+ ve cells (CD62L+ve Helper T cells) |
| CD3+ve CD8+ve | CD62L+ ve cells (CD62L+ve Cytotoxic T cells) |
| CD19+ve cells | CD25+ve cells (CD25+ve B cells) |
| CD19+ve cells | CD62L+ve cells (CD62L+ve B cells) |

**Supplementary figure 1:** Florescence minus one (FMO) gating of peripheral blood mononuclear cells (PBMCs). FMO gating strategy used to identify cells population, like, CD45 positive cells, T lymphocytes (CD3 positive), helper T cells (CD)4 positive, cytotoxic T cells (CD8 positive), B cells (CD19 positive), CD56 and CD16 positive NK cells, CD25 positive helper T cells, CD25 positive cytotoxic T cells, CD62L positive helper T cells, CD62L positive cytotoxic T cells, CD25 positive B cells and CD62L positive B cells.


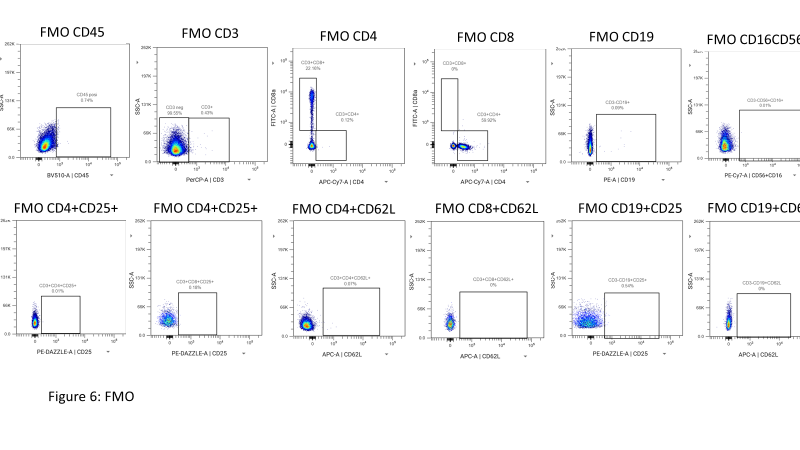


**Supplementary figure 2:** Gating strategy of peripheral blood mononuclear cells (PBMCs) on flowcytometry analysis. to phenotyping different cells. Gating strategy used to identify cells population, like, live and dead cells, CD45 positive cells, T lymphocytes (CD3 positive), helper T cells (CD)4 positive, cytotoxic T cells (CD8 positive), B cells (CD19 positive), CD56 and CD16 positive NK cells, CD25 positive helper T cells, CD25 positive cytotoxic T cells, CD62L positive helper T cells, CD62L positive cytotoxic T cells, CD25 positive B cells and CD62L positive B cells.


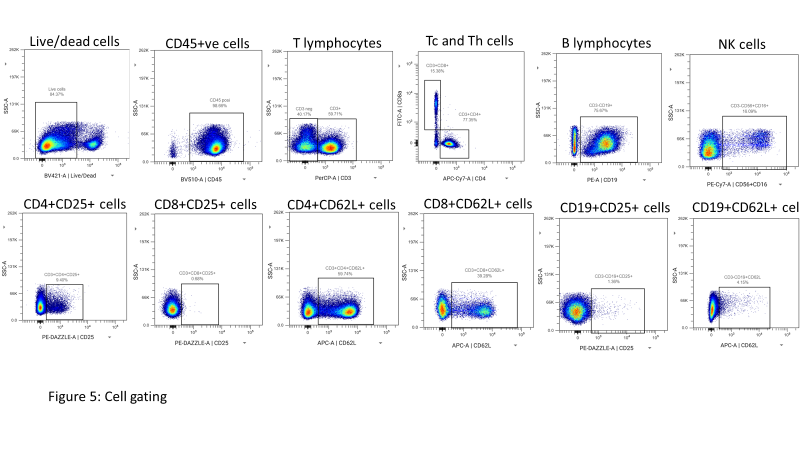

Supplement: Supplementary material — Supplementary fig. 1: Florescence minus one (FMO) gating of peripheral blood mononuclear cells (PBMCs). FMO gating strategy used to identify cells population, like, CD45 positive cells, T lymphocytes (CD3 positive), helper T cells (CD)4 positive, cytotoxic T cells (CD8 positive), B cells (CD19 positive), CD56 and CD16 positive NK cells, CD25 positive helper T cells, CD25 positive cytotoxic T cells, CD62L positive helper T cells, CD62L positive cytotoxic T cells, CD25 positive B cells and CD62L positive B cells. Supplementary fig. 2: Gating strategy of peripheral blood mononuclear cells (PBMCs) on flowcytometry analysis. to phenotyping different cells. Gating strategy used to identify cells population, like, live and dead cells, CD45 positive cells, T lymphocytes (CD3 positive), helper T cells (CD)4 positive, cytotoxic T cells (CD8 positive), B cells (CD19 positive), CD56 and CD16 positive NK cells, CD25 positive helper T cells, CD25 positive cytotoxic T cells, CD62L positive helper T cells, CD62L positive cytotoxic T cells, CD25 positive B cells and CD62L positive B cells. Supplementary table 1: Antibody panel for phenotyping Supplementary table 2: List of all cells population that were quantified as a percentage of the indicated parent population in the panel. [file mmc1.docx]
